# External evaluation of population pharmacokinetic models for tacrolimus in kidney transplant recipients: implications for model-informed precision dosing

**DOI:** 10.1186/s12882-026-05214-3

**Published:** 2026-07-22

**Authors:** Arun Prasath Raju, Priyanka Naithani, Deepesh Kenwar, Sarbpreet Singh, Shiva Kumar SP, Savita Verma Atri, Vivek Kumar, Anjali Agarwal, Ravindra Prabhu Attur, Mahadev Rao, Ashish Sharma, Smita Pattanaik, Surulivelrajan Mallayasamy

**Affiliations:** 1 Department of Pharmacy Practice, Manipal College of Pharmaceutical Sciences, Manipal Academy of Higher Education, Manipal, India; 2 Department of Pharmacology, Postgraduate Institute of Medical Education and Research, Chandigarh, India; 3 Department of Renal Transplant Surgery, Postgraduate Institute of Medical Education and Research, Chandigarh, India; 4 Department of Pediatric Biochemistry, Postgraduate Institute of Medical Education and Research, Chandigarh, India; 5 Department of Nephrology, Postgraduate Institute of Medical Education and Research, Chandigarh, India; 6 Department of Anatomy, Postgraduate Institute of Medical Education and Research, Chandigarh, India; 7 Department of Nephrology, Kasturba Medical College, Manipal Academy of Higher Education, Manipal, India

## Abstract

**Background:**

Tacrolimus, a primary immunosuppressant in kidney transplantation, exhibits variable pharmacokinetics. While model-informed precision dosing (MIPD) using population pharmacokinetic (PopPK) models improve dosing accuracy, models developed in specific populations lack applicability in other populations. We aimed to evaluate the predictive performance of published models integrated in clinical decision support systems (CDSS) in Indian adult renal transplant recipients.

**Methods:**

Predictive performance of four published PopPK models were evaluated using an independent dataset of 120 trough concentrations from 60 adult renal transplant recipients. Models were assessed using prediction based and simulation-based diagnostics. Maximum *a posteriori* (MAP) Bayesian prediction was also used to evaluate the model performance.

**Results:**

A priori predictions (PRED) for all four models demonstrated systematic underprediction, with relative mean prediction error (rME) ranging from − 25.0% to -64.0%, failing clinical acceptability thresholds. However, integrating patient-specific data via MAP Bayesian estimation corrected this bias and improved precision. Notably, all models achieved higher accuracy and precision, with more than 60% of individual predictions falling within $\:\pm\:20\mathrm{\%}$ ($\:{F}_{20}$) of the observed values. Additionally, scaling down the clearance parameter by 30% mitigated the systematic bias observed in the initial a priori predictions.

**Conclusions:**

Published PopPK models exhibited significant systematic bias when applied to Indian renal transplant recipients, posing a substantial risk of overdosing if used without adjustment for initial dose selection. However, these models adapted using MAP Bayesian approach or scaling of clearance parameter demonstrate better predictive performance in this cohort, suggesting they can guide proactive precision dosing in the Indian cohort. Prospective studies evaluating the target attainment and clinical outcomes are required to establish their clinical applicability.

**Supplementary Information:**

The online version contains supplementary material available at https://doi.org/10.1186/s12882-026-05214-3.

## Introduction

Tacrolimus, a potent macrolide immunosuppressant is the primary therapy for preventing graft rejection in solid organ transplantation (SOT) [1, 2]. It has a narrow therapeutic index and highly variable pharmacokinetics (PK), both inter-individual and intra-individual variability that complicates the clinical management [3, 4]. Deviations from the optimal therapeutic range can lead to severe clinical consequences. Underexposure increases the risk of donor-specific antibodies formation, leading to graft rejection. In contrast, overexposure results in severe toxicities, including nephrotoxicity, neurotoxicity, hypertension, post-transplant diabetes mellitus (PTDM), and increased susceptibility to opportunistic infections [5, 6].

The pharmacokinetic variability of tacrolimus can be attributed to multiple sources, including age, body weight, physiological factors such as hematocrit (HCT), serum albumin, hepatic function and genetic factors such as single nucleotide polymorphisms (SNPs) in metabolizing enzymes such as cytochrome P450 3A5 (*CYP3A5*), cytochrome P450 3A4 (*CYP3A4*), their upstream regulators like pregnane x receptor (PXR), P450 oxidoreductase (POR) and drug transporter ATP-binding cassette sub-family B member 1 (*ABCB1*) [2, 5, 7]. The *CYP3A5* genotype significantly affects the clearance of tacrolimus with expressor phenotype (*CYP3A5* *1/*1 or *1/*3) requiring higher doses than non-expressors (*CYP3A5* *3/*3) to achieve therapeutic levels [2, 8, 9]. The distribution of these genetic polymorphisms differ across ethnic groups. *CYP3A5* expression is much higher in African and Asian populations compared to Europeans, leading to higher dose requirements [4, 6, 10]. Factors such as time post-transplant and corticosteroid co-administration also contribute to non-linear kinetics and time-dependent clearance [11].

Therapeutic drug monitoring (TDM) of tacrolimus is routinely performed, by measuring whole-blood trough concentrations ($\:{C}_{0}$) to prevent graft rejection and minimize drug-related toxicity [2, 6]. Current clinical practice mostly follows an empirical dosing approach, where doses are initially prescribed based on body weight (0.1–0.2 mg/kg/day) and subsequently adjusted in a reactive manner based on observed whole blood trough concentration targeting 5–15 ng/mL to meet the recommended therapeutic window [10, 12, 13]. However, this reactive approach fails to account for variables like *CYP3A5* genotype, HCT or changing physiological conditions and requires multiple rounds of reactive dose titrations. This often results in a delay in reaching stable target concentrations, leaving patients vulnerable in the critical early post-transplant period [2]. Furthermore, patients may experience rejection or toxicity even with trough levels within the accepted therapeutic range due to inter-individual pharmacokinetic variability [14, 15]. Model-informed precision dosing (MIPD) has emerged as a superior alternative to address the shortcomings of empirical dosing [16]. MIPD utilizes population pharmacokinetic (PopPK) models combined with maximum *a posteriori* (MAP) Bayesian estimation to individualize therapy, where a patient’s drug concentration data is integrated with population-level priors from the PopPK model to estimate individual PK parameters [2, 17]. This allows for the prediction of the area under the curve (AUC) and future trough concentrations with greater accuracy and precision [8, 17] enabling proactive, individualized dose-selection with minimal dose titration rather than a reactive dose revision with multiple dose titrations.

The MAP Bayesian approach minimizes prediction error by leveraging population information with the individual’s observed data, resulting in the most probable PK parameters for that specific patient [10, 17]. When integrated into clinical decision support systems (CDSS), these tools can guide dose individualization for narrow-therapeutic-index drugs more precisely than empirical dosing approach. Studies have demonstrated that Bayesian forecasting based dosing improves the probability of target concentration attainment (PTA) and reduces variability compared to physician-guided dosing [12, 18, 19], by reducing the time to reach therapeutic targets [11]. The success of a CDSS depends on the underlying PopPK model’s ability to describe the patient population [20]. However, models developed in specific cohorts may not perform well when applied to external clinical populations due to differences in demographics, assay methods, or clinical practices [17, 21]. Systematic external evaluation of published PopPK models is therefore necessary before integrating them into Bayesian forecasting tools for routine clinical care [11]. Validating these models against independent datasets ensures their applicability for precision dosing in real-world settings [22]. Studies have shown that PK models developed in one population may not accurately predict drug behaviour in others due to the ethnic, genetic and environmental differences [22]. Therefore, relying on general dosing guidelines without considering ethnicity-specific genetic distributions can lead to significant dosing errors and highlights the justification for population-specific evaluation [9].

Despite the benefits of model-informed precision dosing (MIPD), there remains a significant gap in its clinical implementation in the Indian transplant scenario. Current practice and research in India predominantly focus on reactive trough-based monitoring or association studies linking specific genotypes such as *CYP3A5* to dose requirements, rather than on the development or validation of comprehensive PopPK models for Bayesian forecasting [23, 24]. Studies involving Indian renal transplant recipients have highlighted the difficulty in achieving stable therapeutic levels using conventional bodyweight-based dosing, which often results in sub-therapeutic exposure in the immediate post-transplant period [25]. Studies in Indian populations have demonstrated significant associations between genetic polymorphisms and tacrolimus dose requirements, underscoring the need for more precise dosing strategies in this cohort [23, 26]. However, the transition to model-based precision dosing has been slow. Given the distinct genotype distribution in the Indian subpopulation compared to Asian or other population cohorts [25, 27], relying on models developed elsewhere without validation or continuing with reactive dosing is a missed opportunity to optimize patient outcomes.

The present study aimed to assess the predictive performance of published tacrolimus population pharmacokinetic models currently implemented in clinical decision‑support systems, using an independent dataset of adult kidney transplant recipients from an Indian tertiary care hospital. Specifically, we evaluated the ability of these models with a MAP Bayesian framework to predict tacrolimus concentrations in an Indian cohort and explored the implications for model‑informed precision dosing and CDSS use in this population.

## Methodology

### Model identification and selection

Population pharmacokinetic models for external evaluation were identified from a recent systematic review exploring various MIPD tools and the models involved [20]. Models were restricted to those currently integrated into CDSS to ensure immediate bedside clinical utility. Models were selected if they were developed primarily using adult kidney transplant recipients, were integrated into at least one MIPD tool and estimation was based on standard parametric approach to ensure reproducibility and comparability. Models developed with rich sampling strategies (* >3* samples per subject) were prioritized. Models were excluded if they were developed exclusively for pediatric populations, estimation was based on iterative 2 stage (IT2S) or non-parametric approaches. Models incorporating specific structural complexities, such as additional compartments for intracellular concentrations were also excluded. A flow chart explaining the screening process is provided in the additional file.

### External data and model evaluation

The independent external dataset was obtained from prior research conducted at Postgraduate Institute of Medical Education and Research, Chandigarh, India (PGIMER) on adult renal transplant recipients. The data was collected in a prospective manner from the patients undergoing renal transplantation. Adult *de novo* renal transplant patients aged 18–60 receiving tacrolimus-based maintenance therapy were included in the study. Whole-blood trough samples ($\:{C}_{0}$) were drawn 15–20 min prior to the morning dose on post operative day 2 and at steady state during month 3. Patients were excluded if they were pregnant or nursing, had acute or chronic infections, undergoing a second or multi-organ transplant. Additionally, individuals who had recently used investigational drugs or medications known to interact with tacrolimus pharmacokinetics such as CYP inducers or inhibitors were excluded. All the patients received tacrolimus-based triple immunosuppressive regimen comprising tacrolimus, mycophenolate mofetil and corticosteroids. Patients were administered immediate-release tacrolimus formulations (Predominantly Pangraf (Panacea Biotec), Tacrograf (Biocon)) twice daily with empiric dose adjustment based on clinician’s judgment. The whole-blood samples for tacrolimus were analysed by LC-MS/MS (ABSiex 4500 Q-Trap). The study was reviewed and approved by the Institutional Ethics Committee (IEC) of PGIMER. Data wrangling and exploratory data analysis was conducted in R (v4.5.1) using the tidyverse package [28, 29].

Predictive performance of the selected models was evaluated using Pumas (v2.7.1) [30]. The selected population pharmacokinetic models were re-implemented with the final model structures, parameter estimates, and covariate parameter relationships reported in the literature. Simulations were performed using the replicated models to generate expected concentration profiles under the original study conditions. Median simulation profiles were extracted from the original publications using WebPlotDigitizer [31] and compared against the simulated median profiles, ensuring that structural parameters and covariate effects were accurately reconstructed prior to external evaluation. Population predictions (PRED) representing expected population concentrations for a typical subject given the covariates were derived using the fixed-effect parameter estimates published for each model. Individual predictions (iPRED) were obtained via MAP Bayesian estimation. Predictions were compared against observed drug concentrations both visually and numerically to identify bias and imprecision.

### Prediction-based diagnostics

Numerical assessment of the model performance was conducted using prediction error metrics such as relative mean prediction error (rME, Eq. 1), relative root mean squared error (rRMSE, Eq. 2 ), and median absolute percentage error (MdAPE, Eq. 3 ) [32]. All the prediction error metrics were calculated as relative percent error rather than absolute error to simplify comparability.

1 $$\:rME\hspace{0.17em}\left({\%}\right)=\frac{1}{N}\sum\:\left(\frac{PRED-OBS}{OBS}\right)\times\:100$$

2 $$\:rRMSE\hspace{0.17em}\left({\%}\right)=\frac{\sqrt{\frac{1}{N}\sum\:{\left(PRED-OBS\right)}^{2}}}{\stackrel{⃐}{OBS}}\times\:100$$

3 $$\:MdAPE\hspace{0.17em}\left({\%}\right)=\mathrm{median}\left(\left|\frac{PRED-OBS}{OBS}\right|\times\:100\right)$$

where, * N* represents the total number of predictions, * PRED* denotes the model-predicted concentration, and * OBS* denotes the observed concentration.

Percentage of predictions falling within ± 30% ($\:{F}_{30}$, Eq. 4 ) and ± 20% ($\:{F}_{20}$, Eq. 5 ) of the observed values were also calculated to assess the overall clinical acceptability of the models.

4 $$\:{F}_{30}\hspace{0.17em}\left({\%}\right)=\frac{\mathrm{count}\left(\left|P{E}_{rel}\right|\le\:0.30\right)}{N}\times\:100$$

5 $$\:{F}_{20}\hspace{0.17em}\left({\%}\right)=\frac{\mathrm{count}\left(\left|P{E}_{rel}\right|\le\:0.20\right)}{N}\times\:100$$

where, $\:P{E}_{rel}$ is the relative prediction error defined as $\:\frac{PRED-OBS}{OBS}$. $\:{F}_{30}$ represents the percentage of predictions falling within $\:\pm\:30{\%}$ of the observed concentrations, and $\:{F}_{20}$ represents predictions falling within $\:\pm\:20{\%}$ of the observed concentrations. Predictive performance of a model was considered to be good when it satisfies the pre-defined benchmarks: rME $\:\pm\:20{\%}$, MdAPE $\:\le\:30{\%}$ and $\:{F}_{30}\ge\:50{\%}$ [11, 33]. As consensus thresholds for rRMSE and $\:{F}_{20}$ are not yet established, they were used as comparative metrics, with lower rRMSE and higher $\:{F}_{20}$ indicating better predictive performance [33].

### Simulation-based diagnostics

Simulation-based diagnostics were performed using the visual predictive check (VPC) and the normalized prediction distribution error (NPDE) to evaluate the model’s ability to reproduce the variability and distribution of the observed external dataset. The VPC was performed by simulating 1,000 replicates of the dataset using the model parameters. To account for heterogeneity, prediction-corrected VPCs (pcVPCs) were also generated by normalizing observed and simulated concentrations to the population predictions. A model was considered to have adequate predictive performance if the observed percentiles fell within the simulation-based 95% CIs, indicating the model correctly characterized the central tendency and variability of the external population.

To assess the full predictive distribution of the models the NPDE metric was used. Under the null hypothesis that the model adequately describes the data, the NPDEs should follow a standard normal distribution with mean 0 and variance 1. The mean deviating from 0 indicates a structural bias. The distribution was assessed using histograms to detect deviations from normality and scatter plots of NPDE versus time and predicted concentrations were examined to identify any systematic trends or model misspecifications over time or concentration ranges.

### Clearance scaling

To investigate the systematic bias identified during the initial predictive performance evaluation, we tested whether an empirical downward adjustment of the population clearance could improve the a priori predictions. The population clearance in each model was iteratively scaled downward by factors ranging from 5% to 50% in increments of 5%. Prediction error metrics were subsequently recalculated for the scaled models and compared against those values of the unadjusted models.

## Results

### Model replication

Four published tacrolimus PopPK models were selected for the evaluation of predictive performance based on the selection strategy (Additional file 1: Fig. S1). Details of the selected models are presented in Table 1, with additional characteristics provided in Additional file 1: Table S1. *CYP3A5* genotype, *CYP3A4* genotype, age, body surface area (BSA), albumin, HCT, serum creatinine (SCr), lean body weight (LBW), race, aspartate aminotransferase (AST), gender and tacrolimus formulation (immediate-release, Prograf^®^; prolonged-release, Advagraf^®^) were the significant covariates affecting the pharmacokinetics in the selected models [34–37]. The key structural features of the selected models along with parameter covariate relationships and random effects are summarized in Table 2. Clearance was affected by HCT, *CYP3A5*, *CYP3A4*, age, albumin, BSA, SCr, race and AST. Volume of distribution (central) is affected by formulation, LBW, Sex, Albumin and AST. Volume of distribution (peripheral) is affected by AST. Transit rate (Ktr) and absorption rate constant (Ka) were affected by formulation. Bioavailability is affected by race, formulation, albumin and AST. All four models were successfully replicated and simulated to match the median profiles from the published studies. Visual comparison graphs are provided in Additional file 1: Figs. S2–S5.

### External data for evaluation

A total of 120 trough concentrations ($\:{C}_{0}$) were included in assessing the performance of the selected models. Trough concentrations were obtained from 60 adult renal transplant recipients at two different occasions, day 2 and month 3 post-transplant. Characteristics of the evaluation cohort are presented in Table 3. The study population was predominantly male (87%, * n=52*) with a mean age of $\:34.8\pm\:7.9$ years. Median tacrolimus trough concentration $\:{C}_{0}$ at day 2 was higher (Median: 18.70 ng/mL, IQR: 15–24) than month 3 (Median: 9.50 ng/mL, IQR: 7.58–10.50). Despite significant shifts in concentration, the dose-normalized concentrations remained relatively stable over time (Day 2 median: 3.71 ng/mL per mg, IQR: 2.98–4.47 and month 3 median: 3.44 ng/mL per mg, IQR: 2.28–4.85).

### Predictive performance of models

**Fig. 1:**
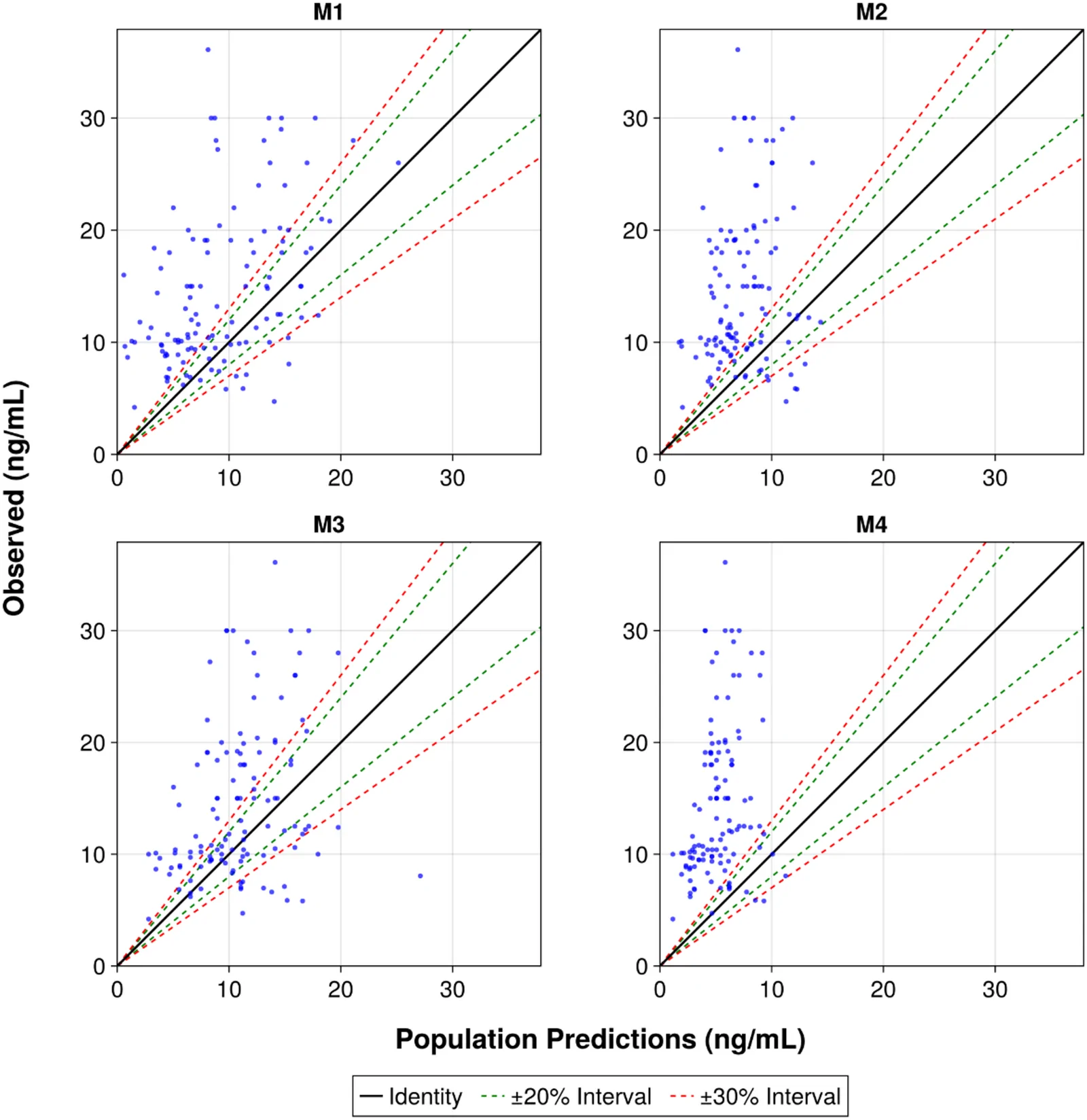
Observed versus predicted tacrolimus trough concentrations for evaluated models

Population predictions (PRED) using typical population parameters revealed a systematic under-prediction and poor precision of tacrolimus concentrations across all four evaluated models in the external cohort. Analysis of the relative mean prediction error (rME) demonstrated negative systematic bias for all four PopPK models, ranging from − 64.0% for the M4 model to -25.0% for the M3 model, this was also confirmed visually using the goodness of fit plots (GoF) in Fig. 1 comparing PRED versus observed concentrations. The detailed individual model diagnostic plots are provided in the Additional file 1: Figs. S6–S9. The weighted residuals (WRES) versus PRED plots reveals a pronounced concentration-dependant bias characterized by a distinct negative slope driven by large positive residuals at lower predictions. Conversely, individual weighted residuals (IWRES) versus individual predictions (iPRED) exhibit a random distribution centered around zero, demonstrating that empirical Bayesian estimation adequately resolves this population-level structural misspecification. The precision of the population predictions was similarly poor, failing to meet clinically acceptable thresholds. MdAPE was highest for M4 at 65.0% and lowest for M3 at 37.0%. Furthermore, rRMSE confirmed the lack of overall accuracy, with values ranging from 53.0% (M3) to 79.0% (M4). This translated into poor clinically acceptable error levels, with the percentage of predictions falling within $\:\pm\:20\mathrm{\%}$ ($\:{F}_{20}$) and $\:\pm\:30\mathrm{\%}$ ($\:{F}_{30}$) of observed values being low. M3 performed best among the models, but achieved only 28.0% $\:{F}_{20}$ and 39.0% $\:{F}_{30}$, whereas M4 showed the lowest accuracy with an $\:{F}_{20}$ of merely 6.7%. Overall, these baseline PRED metrics in Table 4 indicate that relying solely on population parameters is insufficient to capture the ideal pharmacokinetic profile of tacrolimus in this specific patient population.

Across all four candidate models, visual predictive checks (VPCs), and prediction-corrected VPCs (pcVPCs) demonstrated structural misspecification (Additional file 1: Figs. S13–S20). In Models 2 and 3, the observed 90th percentile exceeded the upper boundary of the simulated 95% confidence interval in both the standard VPCs and pcVPCs, confirming systematic under-prediction during the early post-transplant period. The observed percentiles for Models 1 and 4 were largely contained within the simulated confidence intervals, although the pcVPCs indicated a modest tendency toward under-prediction of concentrations during early post-transplant period. These findings were further substantiated by the NPDE analyses (Additional file 1: Figs. S11–S12). Models 2 and 3 showed asymmetric, positively skewed NPDE distributions, with Model 3 additionally exhibiting a pronounced residual cluster at the lower computational limit. Model 4 displayed a distinct bimodal pattern, with residuals clustering across the prediction range, pointing to residual error model inadequacy not detected by the VPC diagnostics alone. Model 1 showed the most symmetric distribution, with only a mild positive skew at lower predictions. These diagnostics demonstrate that the population-level pharmacokinetic behavior of tacrolimus in this cohort was not adequately captured by any of the four candidate models.

Incorporating individual empirical Bayes estimates drastically improved the predictive performance across all models, as reflected in the iPRED metrics as shown in Table 4 (Additional file 1: Fig. S10). The systematic bias was largely corrected where the rME reduced to near-zero values for M3 at -0.6% and M1 at -2.0%, though M2 retained a modest under-prediction bias of -13.0%. Precision was also improved where model M1 exhibited high precision, achieving an MdAPE of just 2.2% and an rRMSE of 3.8%. This high precision in M1 translated into optimal clinically acceptable thresholds, with 100.0% of its individual predictions falling within both the $\:\pm\:20\mathrm{\%}$ ($\:{F}_{20}$) and $\:\pm\:30\mathrm{\%}$ ($\:{F}_{30}$) thresholds. The remaining models (M2, M3, and M4) performed adequately, with MdAPE values clustered between 11.0% and 15.0%, and rRMSE values between 22.0% and 25.0%. Correspondingly, their clinically acceptable thresholds improved, achieving $\:{F}_{20}$ values of 63.0% to 72.0% and $\:{F}_{30}$ values of up to 96.0% for M2.

**Fig. 2:**
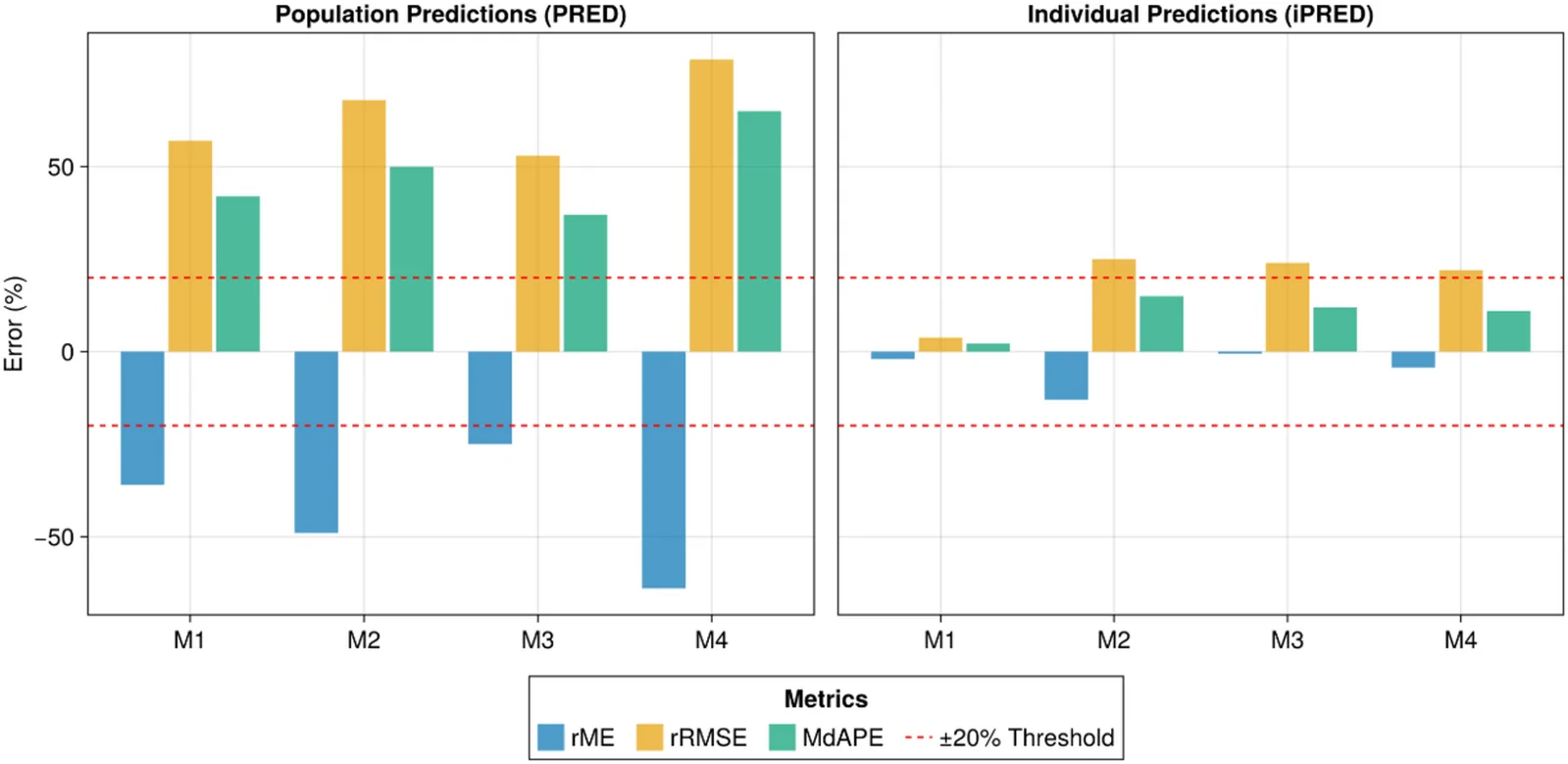
Comparative assessment of predictive performance metrics for evaluated models

**Fig. 3:**
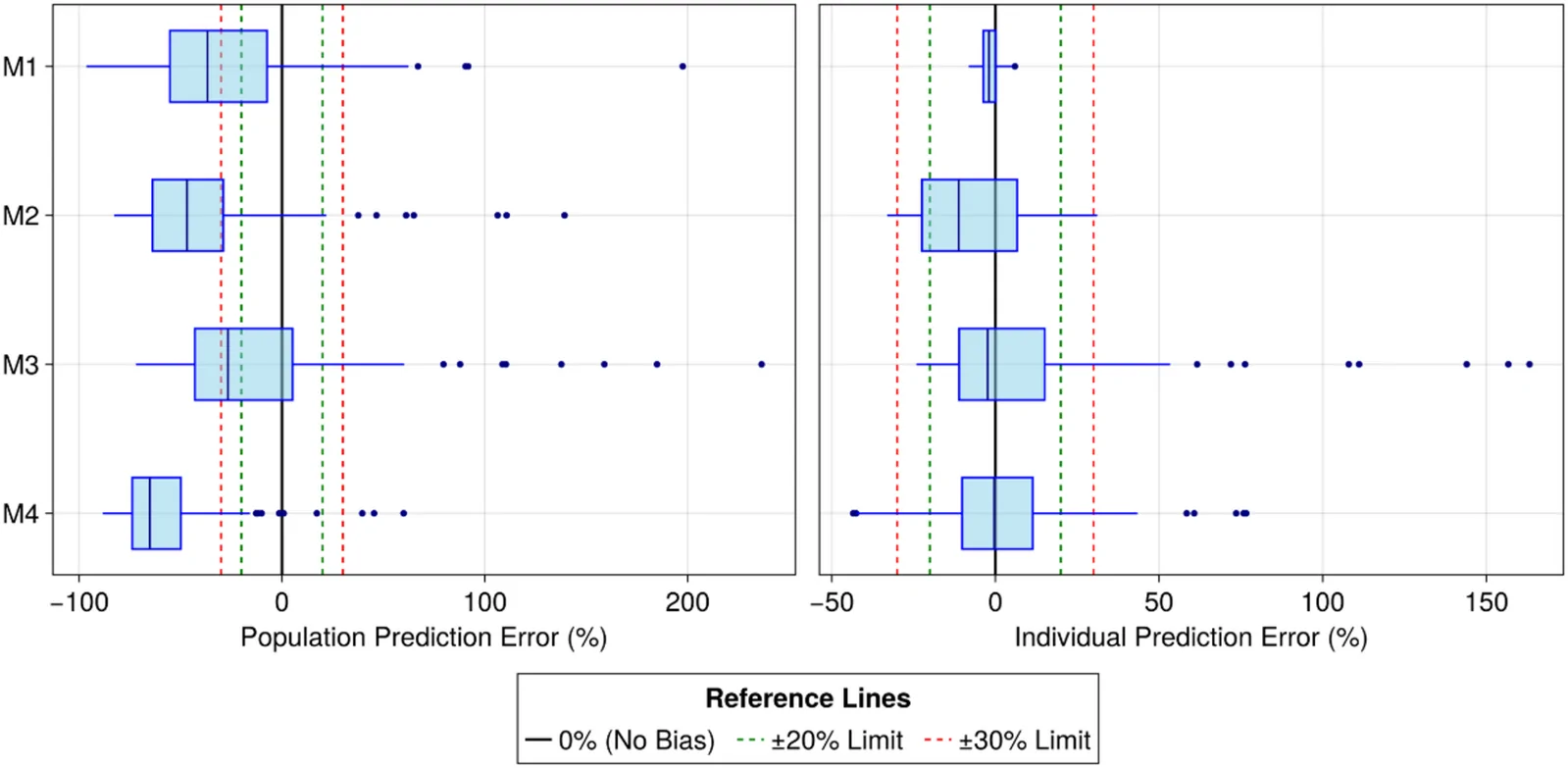
Distribution of prediction errors across the evaluated models. Note: All prediction errors reflect the use of generic immediate-release tacrolimus in the evaluation cohort

While the population-level predictions yielded unacceptable bias, precision, and clinical target attainment across all four models, the integration of patient-specific data mitigated the systematic underprediction and brought the accuracy metrics into clinically useful ranges as shown in Figs. 2 and 3. M3 provided the most robust a priori population predictions and the lowest individual bias, making it a stable structural model. However, M1 demonstrated the highest precision after integrating individual data for MAPB estimation in this population.

### Scaling of clearance parameter

**Fig. 4:**
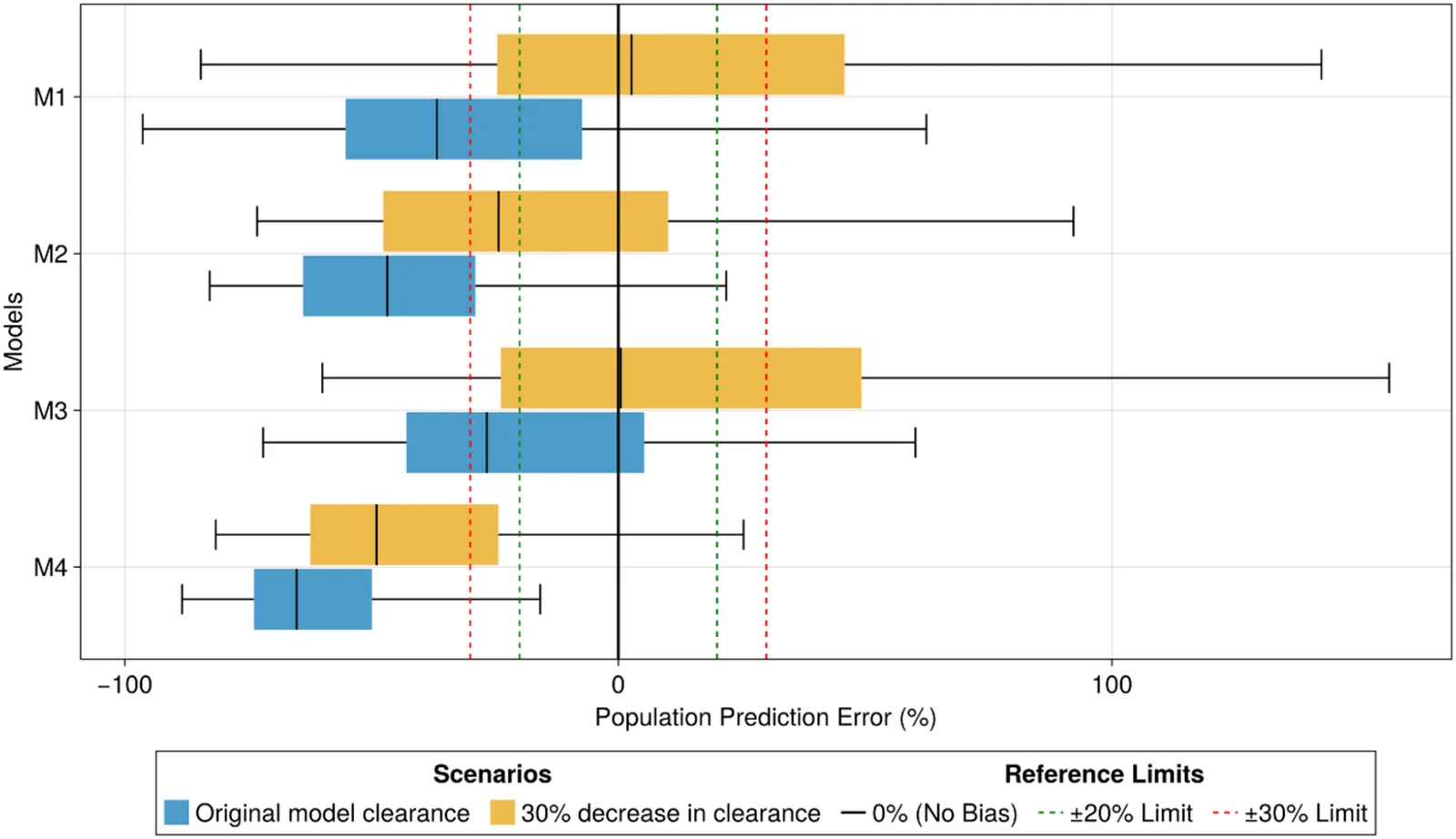
Impact of clearance parameter scaling on population prediction (PRED) bias

The performance of the models was assessed after scaling the clearance parameter downward by 5% to 50% to address the systematic under-prediction. Among the 4 models, 3 of them performed better when the clearance was reduced by 30%. The suboptimal performance of the 4th model may be due to the lack of *CYP3A5* covariate in the final model, which is a significant contributor to the pharmacokinetic variability of tacrolimus. For the M3 model, which initially exhibited a -25.0% rME, the 30% clearance reduction shifted the systematic bias to a clinically negligible margin (6.9%), bringing the typical predictions into better alignment with the observed central tendency of the cohort. Similarly, the M1 model saw its population bias decrease from − 36.0% to 3.3% as shown in Fig. 4. Parameter scaling improved the $\:{F}_{30}$ of the models as summarized in Table 5. M2 demonstrated the most improvement, doubling its $\:{F}_{30}$ from 21.0% in the initial model to 42.0% after the clearance adjustment. M1 also improved, increasing its $\:{F}_{30}$ from 32.0% to 48.0%, while M3 showed a modest increase from 39.0% to 45.0%. M4 remained the least accurate, improving from 9.2% to 22.0%. This improvement emphasizes the use of parameter scaling for safer, more accurate initial dose selection prior to the availability of TDM data. Notably, while the PRED metrics showed improvements due to the clearance reduction, the iPRED metrics remained highly stable and robust.

## Discussion

This study represents the first external evaluation of published population pharmacokinetic (PopPK) models for tacrolimus specifically in an Indian renal transplant cohort. The primary finding of this analysis is the marked discrepancy between the predicted and observed tacrolimus concentrations when utilizing population level priors alone. All four evaluated models (M1–M4) demonstrated negative systematic bias (rME ranging from − 25.0% to -64.0%) and poor precision (rRMSE ranging from 53.0% to 79.0%) when applied to the external Indian dataset. None of the models met the clinically acceptable criteria for a priori prediction, defined as $\:{F}_{30}\ge\:50{\%}$ [11, 38].

The systematic underprediction observed across all models suggests that the clearance of tacrolimus in this Indian cohort is significantly lower, or its bioavailability higher, than in the populations in which these models were initially developed. This aligns with the published literature, where external validations frequently expose the lack of generalizability of models across different ethnicities and clinical practices [11, 39]. The failure of the initial population-level predictions can be traced back to how these models parameterize the biological factors affecting tacrolimus disposition, particularly regarding structural covariates, body composition, and early post-transplant physiology.

The difference in *CYP3A5* allele distribution between ethnic groups is a major determinant of pharmacokinetic variability. While the functional *CYP3A5* expressor phenotype has a frequency of only ~ 5–15% in Western Caucasian populations [2], it is substantially more prevalent in India, where nearly 40–60% of Indian transplant recipients are *CYP3A5* expressors [40–42]. Because this genotype is typically associated with rapid metabolism, one would expect high clearance rates and consequently lower drug concentrations. However, despite accounting for *CYP3A5* status, all evaluated models systematically under-predicted tacrolimus trough concentrations in our cohort. This observation, higher-than-expected concentrations despite a ~ 45% expresser frequency, indicates that the true apparent clearance in the Indian cohort is lower than what the models estimated. This discrepancy suggests that other population-specific factors counteract the *CYP3A5* rapid metabolizer status.

A primary contributor is body composition. The evaluated models were developed using Western cohorts with average body weights between 70 and 80 kg, whereas our Indian cohort averaged only ~ 58 kg. Furthermore, South Asian populations frequently exhibit a “thin-fat” phenotype, characterized by a lower fat-free mass for a given total body weight compared to Caucasians [43]. Because tacrolimus clearance correlates strongly with lean mass and liver volume, models scaling clearance solely by total body weight inherently overestimate metabolic capacity in Indian patients, thus masking the genotype effect. Additionally, formulation-specific differences likely impact relative bioavailability. The original models were developed using innovator formulations (Prograf or Advagraf), whereas our cohort was treated almost exclusively with locally manufactured generic formulations. Minor differences in excipients or dissolution profiles can lead to increased individual bioavailability (* F*) compared to the innovator drug [44, 45]. Because population models estimate *apparent* clearance (* CL/F*), any unmeasured increase in systemic bioavailability directly manifests as a decrease in apparent clearance, contributing heavily to the higher-than-expected concentrations observed in our study. Furthermore, unmeasured pharmacogenetic variation likely contributes to this. The difference in the allele frequency of CYP3A4*22 reduced function varient or ABCB1 polymorphisms reduce the true clearance, counteracting the rapid metabolism of *CYP3A5* expressors [42, 46, 47]. Alongside other unmeasured covariates [38, 48], these factors collectively could explain why unadjusted Western priors overestimate clearance in Indian patients.

The persistent under-prediction of high concentrations observed primarily on postoperative Day 2 suggests additional structural limitations in the evaluated models for capturing early post-transplant physiology. Models developed using data from clinically stable patients (e.g., M1 and M3) lack time-varying clearance functions to describe the transient early postoperative state [49]. During this immediate period, tacrolimus clearance is dynamic; for example, corticosteroid-induced CYP3A4 transcription requires several days to develop, meaning the expected steroid-induced clearance may be incomplete on Day 2 [50]. Concurrent shifts in hematocrit following surgical blood loss alter the fraction of unbound drug available for clearance [51, 52]. Standard structural models omitting these time-varying factors may fail to accurately predict the early postoperative phase.

Despite the poor performance of a priori predictions, this work identifies an improvement in posterior goodness of fit achieved through MAP Bayesian estimation. Once patient-specific concentration data was integrated, the bias was mitigated, and precision metrics improved to clinically useful levels. This confirms that while the population estimates of the published models may not directly fit the Indian population, the structural models and error models effectively describe the intra-individual pharmacokinetics of tacrolimus once the individual parameters are refined. Our results mirror findings from recent external evaluations in other transplant populations. Han et al. found that while published models performed poorly for a priori prediction in lung transplant recipients, Bayesian forecasting with as few as one or two prior observations yielded satisfactory predictive performance [38]. Similarly, Cai et al. demonstrated that Bayesian forecasting significantly improved the robustness of models in liver transplant recipients, overcoming initial predictive failures [11].

In our study, Model M1 showed the best predictive performance in this population due to its higher precision after integrating individual data, whereas Model M3 provided the most robust a priori predictions. From a clinical perspective, M3 may be safer for initial dose calculation if no data is available, while M1 demonstrates better precision using MAP Bayesian estimation. The success of the Bayesian step indicates that these models are structurally “fit-for-purpose” for adaptive dosing [53]. Implementing M1 or M3 into a Bayesian dosing software would likely result in higher target attainment ratios than standard labeling or empirical dosing, particularly after the first TDM sample is available. This supports the transition from reactive empiric approach where dose adjustments are based on trough levels alone to proactive MIPD, which has been shown to increase the proportion of patients within the therapeutic window and reduce time to target concentration [12, 18].

The results of this study suggest that using published models directly from the literature to calculate starting doses poses a risk of overdosing in Indian patients, as evidenced by the negative bias. This highlights the necessity of parameter scaling or re-estimation of baseline population parameters before a model is employed in clinical decision making [54, 55]. Importantly, the 30% reduction in clearance was an empirical adjustment rather than a formal parameter re-estimation used solely to correct the bias observed in our specific dataset and hence lacks universal clinical applicability. While the predictive precision improved, the actual clinical safety and efficacy of relying on these models for ongoing adaptive dosing remains unverified until tested in prospective studies. Until prospective studies are conducted or population specific model is developed the clearance scaled model can be used as a starting-dose guide rather than a substitute for dose optimization and titration with close monitoring.

### Limitations

Few limitations should be considered when interpreting the findings of this study. The external data was from a single center with relatively small sample size, which limits the generalizability of the findings across the highly diverse landscape of the Indian subcontinent. The evaluation was constrained by reliance on sparse sampling, restricted to specifically day 2 and month 3 trough concentrations ($\:{C}_{0}$) which limits the ability to characterize the full PK profile of Tacrolimus. Additionally, the analysis evaluated the models utilizing their originally published covariates without explicitly modeling critical population-specific determinants. Furthermore, unavailable covariates required by the original structural models such as albumin, creatinine, AST, LBW, BSA and CYP3A4 genotype had to be imputed using the mean or median values. Although this imputation strategy was necessary to execute the published models and effectively nullifies the specific covariate’s effect on the predictions, it fails to capture the true inter-individual variability associated with those parameters. Furthermore, all available observations were used simultaneously to derive the empirical Bayes estimates, the improved individual predictions represent the *a posteriori* goodness of fit rather than a true Bayesian forecast. Prospective studies are warranted for full Bayesian forecasting.

## Conclusions

Published population pharmacokinetic (PopPK) models for tacrolimus, primarily developed in other cohorts exhibit significant variability and systematic bias when extrapolated to an Indian renal transplant population. This bias indicates that the baseline clearance in these existing models does not accurately reflect the physiological and pharmacokinetic realities of the Indian population. Specifically, these models over-estimate the clearance likely due to population-specific phenotypic differences that outweigh the expected high-clearance effect of the *CYP3A5* expressor genotype. Hence, utilizing these unadjusted models for initial, a priori dose selection poses a risk of over-dosing and acute toxicity in the immediate post-transplant period. Although a 30% downward scaling of clearance significantly reduced the bias, the models achieved clinically acceptable performance only when adapted via maximum *a posteriori* (MAPB) approach. While the structural framework of the tested models are sound, the population priors are misaligned with the Indian population. Therefore, the implementation of MIPD in the Indian scenario requires prospective studies to validate the adaptive strengths of MAPB for iterative dose adjustment alongside the development of population-specific models or the adoption of localized scaling for optimized patient care.

**Table 1:** Characteristics of selected Tacrolimus PopPK models

Study	Model Identifier	Patients (M/F)	Mean samples	MIPD Integration
Woillard et al. (2011) [34]	M1	38/35 (73 total)	25.5	DoseMeRx, ISBA
Andrews et al. (2019) [35]	M2	204/133 (337 total)	13.4	InsightRx Nova
Benkali et al. (2010) [36]	M3	19/22 (41 total)	12	ISBA
Lu et al. (2019) [37]	M4	276/132 (408 total)	56.8	MWPharm

^*^ **Abbreviations**: M/F: Male/Female; MIPD: Model-Informed Precision Dosing

**Table 2:** Structural, covariate, and variability characteristics of selected Tacrolimus PopPK models

Model	Structural model	Significant covariates	Parameters and covariate relationships	BSV	RUV
M1	Two-compartment with Erlang absorption (3 transit)	*CYP3A5* status, Hematocrit, Formulation	$$\begin{aligned}\:CL/F&=21.2\times\:\left({\left(HCT/35\right)}^{-1.14}\right)\cr&\quad\times\:\left({2.00}^{CYP}\right)\end{aligned}$$	* CL/F*: 28%;$\:{V}_{c}/F$: 31%;$\:{k}_{tr}$: 24%;$\:{V}_{p}/F$: 60%	Prop: 11.3%; Add: 0.71 ng/mL
M2	Two-compartment, 1st-order absorption, linear elimination	*CYP3A5*, *CYP3A4*, Age, BSA, Albumin, SCr, Hematocrit, LBW	$$\begin{aligned}CL/F&=23\times\:\left(1.631\text{ if CYP3A5}\right)\times\:\left(0.8\text{ if CYP3A4*22}\right)\cr&\quad\times\:{\left(Age/56\right)}^{-0.43}\times\:{\left(Albumin/42\right)}^{0.43}\cr&\quad\times\:{\left(BSA/1.93\right)}^{0.88}\times\:{\left(SCr/135\right)}^{-0.14}\cr&\quad\times\:{\left(HCT/0.34\right)}^{-0.76}\end{aligned}$$	* CL/F*: 38.6%;$\:{V}_{1}/F$: 49.2%;$\:{V}_{2}/F$: 53.0%;* Q/F*: 78.7%	Prop: 17.7% (IA), 24.5% (LCMS) Add: 0.88 ng/mL
M3	Two-compartment with Erlang absorption (3 transit cpts)	*CYP3A5*	$$\begin{aligned}\:CL/F&=19\times\:\left(1+1.13\times\:CYP\right)\:\cr&\quad(where\:CYP=1\:for\:expressers)\end{aligned}$$	* CL/F*: 34%;$\:{V}_{1}/F$: 52%;$\:{k}_{tr}$: 51%;$\:{k}_{56}$: 53%	Prop: 8%; Add: 0.7 ng/mL
M4	Two-compartment, 1st-order absorption, lag time	Race, Sex, Albumin (ALB), ln(AST), Formulation	$$\begin{aligned}\:CL/F&=44.3\times\:\left(1+0.59\text{ if Asian}\right)\cr&\quad\times\:{\left(ln\left(AST\right)/3.15\right)}^{-0.318}\end{aligned}$$	* CL/F*: 30.9%;$\:{V}_{c}/F$: 106%;* Q/F*: 39.3%;$\:{V}_{p}/F$: 99%;$\:{k}_{a}$: 35.5%	RV1: 21.1% RV2: 15.8% (log add.)

^*^ **Abbreviations**:* CL/F*: Apparent clearance;$\:{V}_{c}/F$: Central volume of distribution;$\:{V}_{p}/F$: Peripheral volume of distribution;$\:{k}_{tr}$: Transit rate constant;* Q/F*: Inter-compartmental clearance; BSV: Inter-individual variability; RUV: Residual unexplained variability (Prop: Proportional, Add: Additive); BSA: Body Surface Area; HCT: Hematocrit; SCr: Serum Creatinine; LBW: Lean Body Weight; AST: Aspartate Aminotransferase

**Table 3:** Overall patient demographics and clinical characteristics of external cohort

Characteristic	Statistic
Age (years)	34.8 (7.9)
Gender	
Male	52 (87)
Female	8 (13)
CYP3A5 genotype	
* 1/*1	7 (12)
* 1/*3	20 (33)
* 3/*3	33 (55)
Weight (kg)	53.5 (10.8)
Tacrolimus dose (mg)	4.2 (1.7)
Tacrolimus concentration (ng/mL)	14.51 (6.95)
Dose-normalized concentration (ng/mL per mg)	3.79 (1.77)
Hemoglobin (g/dL)	12.2 (1.7)
Hematocrit (%)	37.3 (5.9)

^*^ Statistics presented as mean (standard deviation) for continuous variables and frequency (percentage) for categorical variables

**Table 4:** Summary of predictive performance of models

Prediction.Metrics	M1	M2	M3	M4
PRED (rME) %	-36.0	-49	-25.0	-64.0
iPRED (rME) %	-2.0	-13	-0.6	-4.3
PRED (rRMSE) %	57.0	68	53.0	79.0
iPRED (rRMSE) %	3.8	25	24.0	22.0
PRED (MdAPE) %	42.0	50	37.0	65.0
iPRED (MdAPE) %	2.2	15	12.0	11.0
PRED F₃₀ %	32.0	21	39.0	9.2
iPRED F₃₀ %	100.0	96	86.0	83.0

^*^ **Abbreviations**: PRED: Population prediction; iPRED: Individual prediction; rME: Relative Mean Prediction Error; rRMSE: Relative Root Mean Squared Error; MdAPE: Median Absolute Percentage Error;$\:{F}_{30}$: Percentage of predictions falling within ± 30% of the observed value

**Table 5:** Comparative Performance of Baseline vs. 30% CL-Scaled Models

Model	Baseline PRED (rME %)	Scaled PRED (rME %)	Baseline $\:{\boldsymbol{F}}_{30}$ (%)	Scaled $\:{\boldsymbol{F}}_{30}$ (%)
M1	-36	3.3	32.0	48
M2	-49	-24.0	21.0	42
M3	-25	6.9	39.0	45
M4	-64	-46.0	9.2	22

^*^ **Abbreviations**: PRED: Population prediction; rME: Relative Mean Prediction Error; $\:{F}_{30}$: Percentage of predictions falling within ± 30% of the observed value

## Supplementary Information

Below is the link to the electronic supplementary material.

Supplementary Material 1 : Additional file 1: Supplementary Figures S1–S20 and Table S1 This file contains all supplementary figures, including the model selection flowchart (Fig. S1), model replication and verification plots (Figs. S2–S5), detailed goodness-of-fit diagnostics for each evaluated model (Figs. S6–S9), and comparative individual prediction and normalized prediction distribution error (NPDE) diagnostics (Figs. S10–S12), Visual predictive check and pc vpcs (Figs. S13–S20).

Supplementary Material 2

## Data Availability

The datasets/scripts used and/or analyzed during the current study are available from the corresponding author on reasonable request.

## Abbreviations

PopPK: Population Pharmacokinetics
MIPD: Model-Informed Precision Dosing
TDM: Therapeutic Drug Monitoring
CDSS: Clinical Decision Support Systems
AUC: Area Under the Curve
MAP: Maximum A Posteriori
PRED: Population Prediction
iPRED: Individual Prediction
OBS: Observed Concentration
rME: Relative Mean Prediction Error
rRMSE: Relative Root Mean Squared Error
MdAPE: Median Absolute Percentage Error
$\:{\mathbf{F}}_{20}$: Percentage of predictions within ± 20% of observed value
$\:{\mathbf{F}}_{30}$: Percentage of predictions within ± 30% of observed value
VPC: Visual Predictive Check
NPDE: Normalized Prediction Distribution Error
HCT: Hematocrit
BSA: Body Surface Area
SCr: Serum Creatinine
LBW: Lean Body Weight
AST: Aspartate Aminotransferase
PK: Pharmacokinetics
SOT: Solid Organ Transplantation
SNP: Single Nucleotide Polymorphism
CYP3A5: Cytochrome P450 3A5
CYP3A4: Cytochrome P450 3A4
ABCB1: ATP-binding cassette sub-family B member 1
IT2S: Iterative 2 Stage
PTA: Probability of Target Attainment
PXR: Pregnane X Receptor
POR: P450 Oxidoreductase
