# Supplementary material for: External evaluation of population pharmacokinetic models for tacrolimus in kidney transplant recipients: implications for model-informed precision dosing
Source: BMC Nephrol. 2026 Jul 22;27:618. doi: 10.1186/s12882-026-05214-3 (PMC13678889; doi:10.1186/s12882-026-05214-3)
Supplement: Supplementary file 1 [file 12882_2026_5214_MOESM1_ESM.docx]

External evaluation of population pharmacokinetic models for tacrolimus in kidney transplant recipients: implications for model-informed precision dosing

Additional file 1: Supplementary Figures S1–S10

# Supplementary Figures

Fig. S 1: Model M1 replication and verification. Visual comparison of simulated median profiles against published literature to verify model implementation.

Fig. S 2: Model M2 replication and verification. Visual comparison of simulated median profiles against published literature to verify model implementation.

Fig. S 3: Model M3 replication and verification. Visual comparison of simulated median profiles against published literature to verify model implementation.

Fig. S 4: Model M4 replication and verification. Visual comparison of simulated median profiles against published literature to verify model implementation.

Fig. S 5: Goodness-of-fit diagnostics for Model M1. Includes population prediction (PRED) and individual prediction (iPRED) versus observed concentrations.

Fig. S 6: Goodness-of-fit diagnostics for Model M2. Includes population prediction (PRED) and individual prediction (iPRED) versus observed concentrations.

Fig. S 7: Goodness-of-fit diagnostics for Model M3. Includes population prediction (PRED) and individual prediction (iPRED) versus observed concentrations.

Fig. S 8: Goodness-of-fit diagnostics for Model M4. Includes population prediction (PRED) and individual prediction (iPRED) versus observed concentrations.

Fig. S 9: Observed versus individual predictions (iPRED) for all four evaluated models.

Fig. S 10: Normalized prediction distribution errors (NPDE) versus population predictions (PRED) of evaluated models.
