# Supplementary material for: External evaluation of population pharmacokinetic models for tacrolimus in kidney transplant recipients: implications for model-informed precision dosing
Source: BMC Nephrol. 2026 Jul 22;27:618. doi: 10.1186/s12882-026-05214-3 (PMC13678889; doi:10.1186/s12882-026-05214-3)
Supplement: Supplementary file 2 [file 12882_2026_5214_MOESM2_ESM.docx]

External evaluation of population pharmacokinetic models for tacrolimus in kidney transplant recipients: implications for model-informed precision dosing

Additional file 1: Supplementary Figures S1–S20

# Supplementary Figures

Fig. S 1: Flowchart detailing the selection strategy of published PopPK models for external evaluation.

Fig. S 2: Model M1 replication and verification. Visual comparison of simulated median profiles against published literature to verify model implementation.

Fig. S 3: Model M2 replication and verification. Visual comparison of simulated median profiles against published literature to verify model implementation.

Fig. S 4: Model M3 replication and verification. Visual comparison of simulated median profiles against published literature to verify model implementation.

Fig. S 5: Model M4 replication and verification. Visual comparison of simulated median profiles against published literature to verify model implementation.

Fig. S 6: Goodness-of-fit diagnostics for Model M1. Includes population prediction (PRED) and individual prediction (iPRED) versus observed concentrations.

Fig. S 7: Goodness-of-fit diagnostics for Model M2. Includes population prediction (PRED) and individual prediction (iPRED) versus observed concentrations.

Fig. S 8: Goodness-of-fit diagnostics for Model M3. Includes population prediction (PRED) and individual prediction (iPRED) versus observed concentrations.

Fig. S 9: Goodness-of-fit diagnostics for Model M4. Includes population prediction (PRED) and individual prediction (iPRED) versus observed concentrations.

Fig. S 10: Observed versus individual predictions (iPRED) for all four evaluated models.

Fig. S 11: Normalized prediction distribution errors (NPDE) versus population predictions (PRED) of evaluated models.

Fig. S 12: Normalized prediction distribution errors (NPDE) distribution for all four evaluated models.

Fig. S 13: Visual Predictive Check (VPC) for Model M1.

Fig. S 14: Prediction-Corrected Visual Predictive Check (pcVPC) for Model M1.

Fig. S 15: Visual Predictive Check (VPC) for Model M2.

Fig. S 16: Prediction-Corrected Visual Predictive Check (pcVPC) for Model M2.

Fig. S 17: Visual Predictive Check (VPC) for Model M3.

Fig. S 18: Prediction-Corrected Visual Predictive Check (pcVPC) for Model M3.

Fig. S 19: Visual Predictive Check (VPC) for Model M4.

Fig. S 20: Prediction-Corrected Visual Predictive Check (pcVPC) for Model M4.

# Supplementary Tables

Table S 1: Additional methodological characteristics of the selected PopPK models.

| Study | Model Identifier | Assay Method | CYP3A5 Expressors (%) | Formulation | Sampling schedule |
| --- | --- | --- | --- | --- | --- |
| Woillard et al. (2011) | M1 | TFC-MS/MS | 8.2% | Twice-daily (Prograf®) and Once-daily (Advagraf®) | Prograf: 0–12 h (11 samples) Advagraf: 0–24 h (12 samples) |
| Andrews et al. (2019) | M2 | LC-MS/MS and Immunoassay | 25.5% | Twice-daily immediate-release (Prograf®) | $C_{0}$ trough and AUC curves (0–12 h) |
| Benkali et al. (2010) | M3 | TFC-MS/MS | 12.2% | Once-daily extended-release (Advagraf®) | 0, 0.33, 0.66, 1, 1.5, 2, 3,4, 6, 9, 12, 24 h (12 samples) |
| Lu et al. (2019) | M4 | LC-MS/MS (Immunoassay in 1 study) | Not reported | Immediate-release (Prograf®) and Prolonged-release (Advagraf®) | Multiple samples: 0–24 h |

**Abbreviations:** PopPK: Population pharmacokinetic; LC-MS/MS: Liquid chromatography-tandem mass spectrometry; TFC-MS/MS: Turbulent flow chromatography-tandem mass spectrometry; AUC: Area under the curve; h: hours.
